# Hepatic fascioliasis with metastasis appearance

**DOI:** 10.1590/0037-8682-0117-2023

**Published:** 2023-06-02

**Authors:** Merve Nur Tasdemir, Serdar Aslan, Tumay Bekci

**Affiliations:** 1 Giresun University, Faculty of Medicine, Department of Radiology, Giresun, Turkey.

**FIGURE 1:**
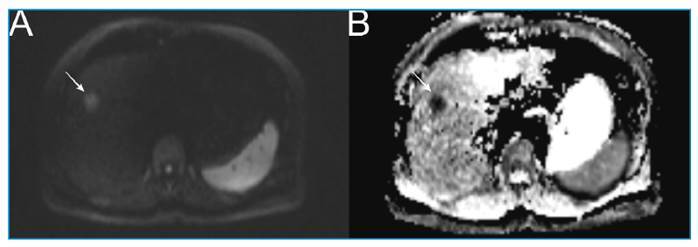
(A-B): Restricted diffusion detected in the lesion on diffusion-weighted images (white arrow).

**FIGURE 2:**
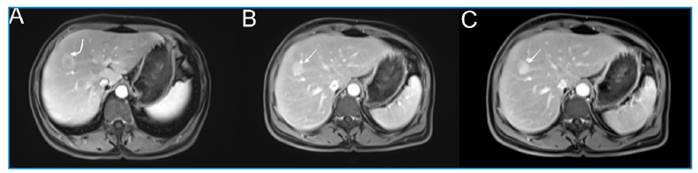
**(A)** Continuous peripheral enhancement observed in the arterial phase (curved arrow). **(B-C)** In the venous and delayed phases, the lesion shows diffuse enhancement (white arrow).

A 59-year-old woman with right upper quadrant abdominal pain was admitted to the Department of Internal Medicine. Her ultrasonography revealed a hyperechoic liver lesion, while her laboratory test results were normal. Magnetic resonance imaging was performed, which revealed restricted diffusion in the lesion on diffusion-weighted images (Figure 1). We observed continuous peripheral enhancement during the arterial phase of the lesion and diffuse enhancement in the venous and delayed phases (Figure 2). Metastases and atypical hemangiomas were considered differential diagnoses. The histopathological findings were consistent with those of hepatic fascioliasis. 

Hepatic fascioliasis has nonspecific clinical symptoms^1^. There is a lack of information in the literature regarding the imaging features of hepatic fascioliasis^2^ ^-^ ^3^. Imaging has become increasingly useful in diagnosing and treating infectious liver diseases^3^. Noninvasive imaging techniques are required to differentiate fascioliasis lesions from other focal liver lesions. Hepatic fascioliasis extends to the subcapsular area and thickens the liver capsule^1^ ^-^ ^3^. The lesions show peripheral enhancement after contrast administration on magnetic resonance imaging^3^.

In this case, diffuse enhancement was observed instead of peripheral enhancement, and there was no tract extending into the subcapsular region. No clinical history was suggestive of parasitic infections.

Misdiagnosing hepatic fascioliasis as metastasis may result in a dearth of treatment. It is necessary to report the imaging findings of hepatic fascioliasis. Noninvasive techniques must be used more frequently in the management of hepatic fascioliasis.
